# Glycolysis Metabolites and Risk of Atrial Fibrillation and Heart Failure in the PREDIMED Trial

**DOI:** 10.3390/metabo11050306

**Published:** 2021-05-11

**Authors:** Nerea Becerra-Tomás, Miguel Ruiz-Canela, Pablo Hernández-Alonso, Mònica Bulló, Jun Li, Marta Guasch-Ferré, Estefanía Toledo, Clary B. Clish, Ramon Estruch, Emilio Ros, Montserrat Fitó, Chih-Hao Lee, Kerry Pierce, Fernando Arós, Lluís Serra-Majem, Liming Liang, Cristina Razquin, Enrique Gómez-Gracia, Miguel A. Martínez-González, Frank B. Hu, Dolores Corella, Jordi Salas-Salvadó

**Affiliations:** 1 Department of Preventive Medicine, University of Valencia, 46010 Valencia, Spain; nerea.becerra@uv.es (N.B.-T.); dolores.corella@uv.es (D.C.); 2 Universitat Rovira i Virgili, Departament of Biochemistry and Biotechnology, Human Nutrition Unit, 43201 Reus, Spain; 3 Institut d’Investigació Sanitària Pere Virgili (IISPV), 43204 Reus, Spain; monica.bullo@urv.cat; 4 Consorcio Centro de Investigaciones Biomédicas en Red, M.P (CIBEROBN), Institute of Health Carlos III (ISCIII), 28029 Madrid, Spain; mcanela@unav.es (M.R.-C.); etoledo@unav.es (E.T.); restruch@clinic.cat (R.E.); eros@clinic.cat (E.R.); mfito@imim.es (M.F.); aborau@secardiologia.es (F.A.); lluis.serra@ulpgc.es (L.S.-M.); crazquin@unav.es (C.R.); mamartinez@unav.es (M.A.M.-G.); 5 Department of Preventive Medicine and Public Health, University of Navarra, 31008 Pamplona, Spain; 6 IdiSNA, Navarra Institute for Health Research, 31008 Pamplona, Spain; 7 Department of Endocrinology and Nutrition, Virgen de la Victoria University Hospital, University of Malaga (IBIMA), 29010 Malaga, Spain; 8 Department of Biochemistry and Biotechnology, Faculty of Medicine and Health Sciences, University Rovira i Virgili (URV), 43201 Reus, Spain; 9 Department of Nutrition, Harvard TH Chan School of Public Health, Boston, MA 02115, USA; junli@hsph.harvard.edu (J.L.); mguasch@hsph.harvard.edu (M.G.-F.); nhbfh@channing.harvard.edu (F.B.H.); 10 Department of Epidemiology, Harvard TH Chan School of Public Health, Boston, MA 02115, USA; 11 Channing Division for Network Medicine, Department of Medicine, Brigham and Women’s Hospital and Harvard Medical School, Boston, MA 02115, USA; 12 The Broad Institute of Harvard and MIT, Boston, MA 02142, USA; clary@broadinstitute.org (C.B.C.); kpierce@broadinstitute.org (K.P.); 13 Department of Internal Medicine, Institut d’Investigacions Biomèdiques August Pi Sunyer (IDIBAPS), Hospital Clinic, University of Barcelona, 08036 Barcelona, Spain; 14 Lipid Clinic, Department of Endocrinology and Nutrition, IDIBAPS, Hospital Clinic, University of Barcelona, 08036 Barcelona, Spain; 15 Cardiovascular and Nutrition Research Group, Institut de Recerca Hospital del Mar, 08036 Barcelona, Spain; 16 Department of Molecular Metabolism, Harvard TH Chan School of Public Health, Boston, MA 02132, USA; clee@hsph.harvard.edu; 17 Department of Cardiology, University Hospital of Alava, 01009 Vitoria, Spain; 18 Research Institute of Biomedical and Health Sciences (IUIBS), University of Las Palmas de Gran Canaria and Service of Preventive Medicine, Complejo Hospitalario Universitario Insular Materno Infantil (CHUIMI), Canary Health Service, 35001 Las Palmas de Gran Canaria, Spain; 19 Department of Biostatistics, Harvard TH Chan School of Public Health, Boston, MA 02115, USA; lliang@hsph.harvard.edu; 20 Department of Preventive Medicine and Public Health, School of Medicine, University of Málaga, 29010 Málaga, Spain; egomezgracia@uma.es; 21 Nutrition Unit, University Hospital of Sant Joan de Reus, 43204 Reus, Spain

**Keywords:** glycolysis, heart failure, atrial fibrillation, PREDIMED study

## Abstract

The increased prevalence of atrial fibrillation (AF) and heart failure (HF) highlights the need to better understand the mechanisms underlying these cardiovascular diseases (CVDs). In the present study, we aimed to evaluate the association between glycolysis-related metabolites and the risk of AF and HF in a Mediterranean population at high risk of CVD. We used two case–control studies nested within the PREDIMED trial. A total of 512 incident AF cases matched to 734 controls, and 334 incident HF cases matched to 508 controls, were included. Plasma metabolites were quantified by using hydrophilic interaction liquid chromatography coupled with high-resolution negative ion mode MS detection. Conditional logistic regression analyses were performed. The results showed no association between baseline plasma glycolysis intermediates and other related metabolites with AF. Only phosphoglycerate was associated with a higher risk of HF (OR _for 1 SD increase_: 1.28; 95% CI: 1.06, 1.53). The present findings do not support a role of the glycolysis pathway in the pathogenesis of AF. However, the increased risk of HF associated with phosphoglycerate requires further studies.

## 1. Introduction

Heart failure (HF) and atrial fibrillation (AF)—the most common type of arrhythmia—have emerged as major cardiac public health problems. The increasing prevalence of both conditions is associated with both increased morbidity and mortality [1,2]. HF and AF often coexist, and the two dysfunctions share many common risk factors including age, obesity, type 2 diabetes (T2D), and hypertension [3]. However, the traditional risk factors do not completely explain all AF and HF cases, and a deeper knowledge of the pathophysiology of HF and AF is needed [4,5]. In this sense, metabolomics could enhance our understanding of their pathogenic pathways, and help develop preventive strategies through the identification of novel risk biomarkers for these complex diseases.

In patients with AF and HF, perturbations of the glycolysis metabolism have been detected [6,7]. Nevertheless, it is still debated whether altered glycolysis metabolism happens and is implicated before the development of AF and HF, or whether it is a consequence of these diseases [8].

So far, prospective metabolomic studies on AF and HF have focused on a broad range of metabolites involved in various pathways, and just a few have also included glycolysis-related metabolites. In the Framingham Heart Study, sucrose, pyruvate, glucose/fructose/galactose, phosphoglycerate, α-glycerophosphate, phosphoenolpyruvate, and lactate intermediate glycolysis metabolites were not significantly associated with an increased risk of AF [9]. Similarly, in the Atherosclerosis Risk in Communities (ARIC) study, no association was observed between glycerol 3-phosphate, lactate, and AF incidence [10]. Regarding HF, the evidence is even more limited. Only the ARIC study has evaluated the association between plasma lactate and incident HF, showing an increased risk in those individuals located in the highest quartile compared to those in the lowest [11]. As a result, current knowledge about the association between intermediate metabolites of the glycolysis pathway and the development of HF and AF is scarce and inconclusive.

Our aim was to examine associations between baseline glycolysis intermediates (3-phosphoglycerate, hexose monophosphate, fructose/glucose/galactose, lactate, sucrose, α-glycerophosphate, phosphoenolpyruvate, and the ratio of phosphoenolpyruvate to lactate) and other related metabolites (hexose monophosphate and sucrose) and the risk of incident AF and HF in the PREDIMED study.

## 2. Results

Table 1 depicts the baseline characteristics of the study population for the two case–control studies. AF cases were more likely to have hypertension, HF cases were more likely to present diabetes, and both had higher BMI compared with controls.

### 2.1. Baseline Glycolysis Intermediate Metabolites, Other Related Metabolites, and Risk of AF

Table 2 shows the association between baseline plasma metabolites and the risk of AF incidence. Overall, no significant associations were observed between any of the metabolites analyzed and AF.

No significant interactions were observed except for the association between α-glycerophosphate and AF, according to the intervention groups. The matched OR and 95% CI per 1-SD increment in the control group was 1.48 (1.11, 1.99), whereas no significant association was observed in the intervention (MedDiet+EVOO and MedDiet+mixed nuts combined) group.

### 2.2. Baseline Glycolysis Intermediate Metabolites, Other Related Metabolites, and Risk of HF

Associations between baseline plasma metabolites and HF risk are shown in Table 3. In the fully adjusted model, we only observed a significant association per one SD increment in levels of phosphoglycerate (OR _for 1 SD increase_: 1.28; 95% CI: 1.06, 1.53), whereas no association was observed when this metabolite was modeled as quartiles. Fructose/glucose/galactose metabolites were associated with an increased risk of HF in the crude model (adjusted for age, sex, and recruitment center by the matching approach of controls) when modeled as continuous (OR _for 1 SD increase_: 1.17: 95% CI: 1.01, 1.35), but this association did not reach statistical significance after multivariate adjustment. Similarly, sucrose was significantly associated with higher risk of HF when modeled as both quartiles (OR: 1.92; 95% CI: 1.26, 2.94 for Q4 vs. Q1) and continuous (OR _for 1 SD increase_: 1.26; 95% CI: 1.08, 1.47) in the raw analysis, but the association disappeared in the multivariate model.

We found a significant statistical interaction (*p*-value < 0.05) between the intervention group (MedDiet groups merged vs. control group) and phosphoglycerate and sucrose when modeled as continuous. The risk of AF was higher in participants allocated to the control group (OR _for 1 SD increase_: 1.67; 95% CI: 1.21, 2.30, and OR _for 1 SD increase_: 1.48; 95% CI: 1.11, 1.99 for phosphoglycerate and sucrose, respectively). We also observed a significant statistical interaction with T2D for phosphoglycerate, showing a higher risk of AF in those individuals with T2D (OR _for 1 SD increase_: 1.57; 95% CI: 1.24, 1.98). No other interactions were observed between baseline metabolites, the intervention group, and T2D.

### 2.3. Baseline Glycolysis Intermediate Metabolites, Other Related Metabolites, and Risk of AF and HF by Diabetes Status

Associations between baseline plasma metabolites and AF and HF risk by diabetes status are shown in Table 4. No associations were observed between a 1-SD increase in any of the metabolites and AF by diabetic status. Regarding HF risk, only a significant positive association was observed between a 1-SD increase in phosphoglycerate in participants with diabetes (OR _for 1 SD increase_: 1.57; 95% CI: 1.24, 1.99). No other significant associations were observed.

## 3. Discussion

The results of the present analysis, which included two case–control studies nested within the PREDIMED trial, showed no association between plasma glycolysis and related metabolites and AF risk. Phosphoglycerate was the only metabolite associated with a higher risk of HF when modeled as a continuous variable. Of note, interactions between phosphoglycerate and the intervention group and T2D status were found, showing that the adverse effect of this metabolite on HF was limited to the control group and individuals with T2D.

Previous studies have reported glucose oxidation impairment and increased glycolysis activity in patients with HF, which is reflected by higher levels of pyruvate and/or lactate compared to healthy controls [8,12,13,14]. It has been suggested that these changes in myocardial metabolism may precede cardiac anatomical modifications that lead to heart failure [15]. However, it is still unclear if altered glycolysis metabolic profiling could help to identify those patients at high risk for AF and HF.

To the best of our knowledge, this is the first study specifically conducted to evaluate the association between baseline glycolysis intermediates and other related metabolites and the risk of AF and HF. In agreement with our findings, in the Framingham Heart Study, plasma sucrose, phosphoglycerate, α-glycerophosphate, PEP, pyruvate, glucose/fructose/galactose, and lactate were unassociated with AF development [9]. Similarly, no associations between serum glycerol 3-phosphate, lactate, and glucose identified through a non-targeted metabolomic approach and the risk of AF were reported in the ARIC study [10]. Although these results suggest that glycolysis intermediate metabolites do not influence the development of AF, the paucity of studies in this field does not allow us to draw solid conclusions.

Regarding HF, studies are even scarcer. To the best of our knowledge, only one metabolomic study, conducted in the framework of the ARIC study, has analyzed the association between plasma lactate and incident HF, showing that individuals in the highest quartile had a 35% increased risk of HF development compared to those in the lowest quartile [11]. The discrepancies in our results could be due to the method used to analyze lactate. In the ARIC study plasma lactate was analyzed using an enzymatic reaction to convert lactate to pyruvate. However, in the present study, lactate was measured using hydrophilic interaction liquid chromatography, coupled with high-resolution negative ion mode mass spectrometry detection. Of note, the higher risk of HF associated with phosphoglycerate observed in the present analysis deserves further study since, as far as we know, this is the first time that this association has been reported. Importantly, serum uric acid—a putative modulator of carbohydrate and lipid metabolism [16]—has been recently found to be an independent risk factor for both HF and fatal HF in an Italian cohort study of more than 20,000 subjects [17].

The present study has some limitations that should be considered. First, the study participants comprised elderly individuals at high risk of CVD, making the generalization of the findings difficult. Second, we do not have information for pyruvate, the end product of glycolysis, because it was one of the metabolites most susceptible to column degradation during the HILIC-neg analyses and did not pass the quality control process. However, to overcome this important limitation, we calculated the PEP to lactate ratio, so as to have an indirect measure of pyruvate. Third, we did not adjust our analyses for HF for the use of diuretics, which is common in these patients. Fourth, although metabolomics have the advantage of detecting a broad range of metabolites that can have important clinical utility in relation to disease prediction, blood and urine metabolites cannot fully inform us about the metabolic changes occurring only in the myocardium or any particular organ since, for this purpose, the analysis of changes in metabolites across the organ must be assessed [18]. Participants included in the current study presented other comorbidities, such as obesity or type 2 diabetes, which are diseases accompanied by pathological changes in multiple systems and organs, and previously associated with glycolysis-related metabolites [19]. This fact could contribute to the plasma metabolomics profile obscuring the true association between glycolysis intermediate metabolites and AF and HF risk.

## 4. Materials and Methods

### 4.1. Study Design and Participants

The PREDIMED study (ISRCTN35739639) was a randomized, multicenter, parallel- group clinical trial conducted in Spain to evaluate the effectiveness of two Mediterranean diets (one supplemented with extra virgin olive oil and the other with mixed nuts) on the primary prevention of cardiovascular disease (CVD), compared to a low-fat control diet. The design and methods of the PREDIMED study can be found elsewhere (PREDIMED website: http://www.predimed.es (accessed on 1 April 2021), and [20]). Briefly, 7447 elderly participants aged 55–80 years at high risk of CVD were recruited, from 2003 to 2009, and allocated to one of the three possible intervention groups: MedDiet supplemented with EVOO, MedDiet supplemented with mixed nuts, and low-fat control group. Eligible participants were men and women free from CVD at baseline who reported either T2D or at least three major risk factors including smoking, elevated LDL cholesterol levels, low HDL cholesterol levels, hypertension, overweight or obesity, or a family history of premature coronary heart disease.

**Figure 1:**
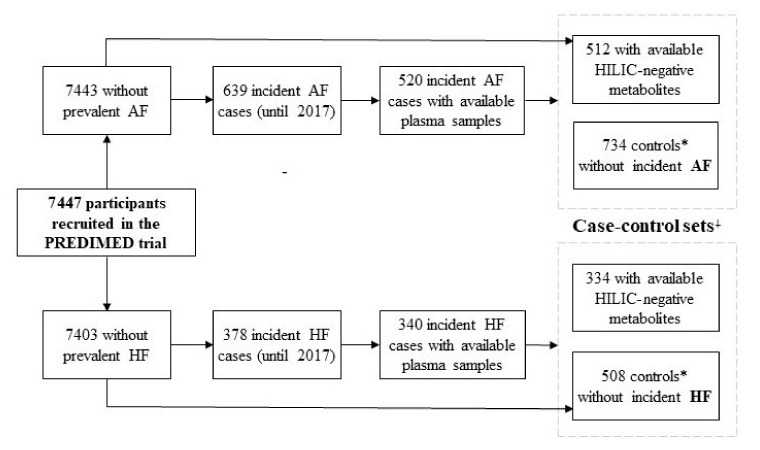
Flowchart of the study subjects. * Incidence density sampling with replacement was used as the control sampling method. ⸸ Case–control sets matched by age, sex, and recruitment center. Abbreviations: AF: atrial fibrillation; HF: heart failure; HILIC: hydrophilic interaction liquid chromatography; PREDIMED: Prevención con Dieta Mediterránea.

For the present analysis, we used two case–control studies nested within the PREDIMED trial. Figure 1 shows the flowchart for both case–control studies. A total of 512 and 334 incident cases of AF and HF were ascertained, respectively, after excluding prevalent cases, incident cases without plasma samples, and cases without HILIC-negative metabolites. We selected matched controls by using the incidence density sampling with replacement [21]. This method involves randomly matching each case to a sample of all of those participants who are at risk at the time of the occurrence of the incident case. Selected controls could be sampled again as a control for future cases, and may later become cases themselves [22]. One to three controls per case were matched by year of birth (±5 years), sex, and recruitment center. The number of controls was 734 and 508 for AF and HF, respectively (Figure 1).

### 4.2. Sample Collection and Metabolomic Analysis

Participants provided fasting blood samples at their baseline visits, which were processed to obtain plasma and stored at −80 °C at each recruitment center until analysis. In order to reduce bias and inter-assay variability, samples from case–control pairs were randomly sorted and analyzed in the same batch. Intermediate glycolysis metabolites—phosphoglycerate (HMDB0000807), fructose/glucose/galactose (HMDB0000122), lactate (HMDB0000190), α-glycerophosphate (HMDB0000126), and phosphoenolpyruvate (HMDB0000263)—and other related metabolites—hexose monophosphate (HMDB0000124) and sucrose (HMDB0000258)—were measured using hydrophilic interaction liquid chromatography coupled with high-resolution negative ion mode mass spectrometry detection (HILIC-neg), as previously described [23,24]. Specifically, HILIC analyses of water-soluble metabolites in the HILIC-neg mode were conducted using an LC–MS system composed of an ACQUITY UPLC system (Waters) and a QTRAP 5500 mass spectrometer (SCIEX). Plasma samples (30 μL) were prepared via protein precipitation, with the addition of 4 volumes of 80% methanol containing inosine-15N4, thymine-d4, and glycocholate-d4 internal standards (Cambridge Isotope Laboratories). Samples were centrifuged for 10 min at 9000× *g*, maintaining a stable temperature of 4 °C, and the supernatants were injected directly into a 150 × 2.0 mm Luna NH2 column (Phenomenex). The column was eluted at a flow rate of 400 μL/min with initial conditions of 10% mobile phase A (20 mmol/L ammonium acetate and 20 mmol/L ammonium hydroxide in water) and 90% mobile phase B (10 mmol/L ammonium hydroxide in 75:25 *v/v* acetonitrile/methanol), followed by a 10-min linear gradient to 100% mobile phase A. MS analyses were performed using electrospray ionization and selective multiple reaction monitoring scans in the negative ion mode. To enable assessment of data quality, and to facilitate data standardization across the analytical queue and sample batches, pairs of pooled plasma reference samples were analyzed at intervals of 20 study samples. One sample from each pair of pooled references served as a passive quality control sample to evaluate the analytical reproducibility for measurement of each metabolite, while the other pooled sample was used to standardize using a “nearest neighbor” approach, as previously described [25]. Standardized values were calculated using the ratio of the value in each sample over the nearest pooled plasma reference multiplied by the median value measured across the pooled references.

Pyruvate (HMDB0000243) was one of the metabolites most susceptible to column degradation during the HILIC-neg analyses, and did not pass the quality control process. Therefore, information about this metabolite was not included in the present analyses. Similarly, hexose diphosphate (HMDB0001058) was not analyzed because of the high amount of missing values (>60%). We also calculated the PEP to lactate ratio. All metabolomic analyses were performed at the Broad Institute of MIT & Harvard.

### 4.3. Outcome Assessment

AF and HF were considered to be secondary endpoints in the PREDIMED trial protocol (a composite of non-fatal myocardial infarction, stroke, and cardiovascular disease death being the primary endpoint). In the present analysis we considered all of the incident cases diagnosed between 2003 and December 2017. Participants from one recruitment center were censored at December 2014 to be selected as controls, since in this center the follow-up was stopped prematurely.

Physicians blinded to the intervention group collected information related to these outcomes from continuous contact with participants and primary health care physicians, yearly follow-up visits, and annual ad hoc reviews of medical charts and consultation of the National Death Index. When a clinical diagnosis of AF or HF was made, the corresponding clinical records of hospital discharge, outpatient clinics, and family physicians’ records were collected. The medical charts were codified with an alphanumeric code and sent anonymously to the clinical endpoint adjudication committee, which adjudicated the events according to prespecified criteria. All relevant documents were independently appraised by two cardiologists, and any disagreement on the classification of the event was solved by contacting a third cardiologist (the committee’s chair). In some cases, further data were demanded in order to complete the adjudication.

AF cases were identified from an annual review of all medical records of each participant, and annual electrocardiograms (ECG) performed at yearly follow-up visits. When AF was present in the ECG or quoted anywhere in the medical reports, the relevant documents were sent to the clinical endpoint committee for their evaluation. We did not include AF events associated with myocardial infarction or cardiac surgeries.

HF diagnosis was made in accordance with the 2005 guidelines of the European Society of Cardiology [26].

### 4.4. Covariates Assessment

At baseline, a general questionnaire about lifestyle, medical history, educational level, medication use, and previous history of diseases was administered to all participants. The Spanish version of the Minnesota Leisure Time Physical Activity Questionnaire [27] was used to assess physical activity. Trained study personnel took anthropometric measurements, and BMI was estimated as weight divided by height squared (kg/m^2^).

### 4.5. Statistical Analysis

We normalized and scaled all individual metabolites in multiples of 1 SD using Blom’s inverse normal transformation [28]. The baseline characteristics of the study population were presented for cases and controls expressed as mean ± SD for quantitative traits and n (%) for categorical variables. The participants were divided into quartiles of intermediate glycolysis metabolites, with cut-off points estimated according to the distribution of metabolites among the controls (participants who did not develop HF or AF during the follow-up). Matched odds ratios (OR) and their 95% confidence intervals (CIs), considering the first quartile as the reference category, were calculated using conditional logistic models, which considered the matching between cases and controls. We also calculated the matched ORs for 1-SD increments of each metabolite, including them as continuous variables. We performed crude models (matched by sex, age, and recruitment center) and multivariate models adjusted for the intervention group (MedDiet vs. control group), body mass index (kg/m^2^), smoking (current, former, or never), leisure time physical activity (metabolic equivalent tasks in min/d), prevalent chronic conditions at baseline (dyslipidemia, hypertension, and T2D), family history of coronary heart disease, education level (primary or lower/secondary or higher), and medication for dyslipidemia, hypertension, and T2D. The test for interactions between individual metabolites as continuous (per 1-SD increment) and the intervention group (both MedDiet groups combined vs. the control group) or T2D prevalence was performed by means of likelihood ratio tests.

All statistical procedures were performed using R v. 3.6.3 statistical software, and a two-sided P-value of less than 0.05 was considered significant.

## Figures and Tables

**Table 1:** Baseline participant characteristics of heart failure (HF) and atrial fibrillation (AF) incident cases and controls.

	Atrial Fibrillation				Heart Failure	
Variables	Controls (n = 545)	Cases (n = 512)	*p*-Value	Controls (n = 408)	Cases (n = 334)	*p*-Value
Age (years)	68.39 (6.21)	68.28 (6.07)	0.772	70.12 (5.92)	70.34 (5.90)	0.615
Women (%)	271 (49.7)	255 (49.8)	1	224 (54.9)	196 (58.7)	0.337
BMI (kg/m^2^)	29.71 (3.7)	30.67 (3.8)	<0.001	29.24 (3.6)	31.07 (3.8)	<0.001
LTFA (METs-min/d)	231.2 (223.0)	228.1 (216.6)	0.823	212.9 (218.6)	216.1 (203.1)	0.837
Systolic blood pressure (mmHg)	146 (19)	149 (22)	0.239	145 (20)	151 (20)	0.009
Diastolic blood pressure (mmHg)	82 (10)	82 (12)	0.765	80 (10)	81 (11)	0.812
Fasting glucose (mg/dL)	120.5 (39.0)	121.8 (44.3)	0.631	121 (40)	130 (52)	0.011
LDL cholesterol (mg/dL)	130.5 (31.5)	128.1 (34.4)	0.255	128.6 (34.4)	127.2 (34.8)	0.591
Hypertension (%)	447 (82.0)	452 (88.3)	0.006	335 (82.1)	293 (87.7)	0.045
Dyslipidemia (%)	382 (70.1)	332 (64.8)	0.079	281 (68.9)	214 (64.1)	0.193
T2D (%)	278 (51.0)	245 (47.9)	0.335	212 (52.0)	197 (59.0)	0.066
Family history of premature CHD (%)	110 (20.2)	98 (19.1)	0.727	0.46 (1.54)	0.52 (1.69)	0.645
Oral hypoglycemic agents (%)	117 (32.5)	156 (30.5)	0.525	134 (32.8)	134 (40.1)	0.048
Lipid-lowering agents (%)	225 (41.3)	207 (40.4)	0.826	166 (40.7)	133 (39.8)	0.870
Antihypertensive agents (%)	110 (20.2)	143 (27.9)	0.004	96 (23.5)	85 (25.4)	0.603
Other medication use (%)	137 (25.1)	152 (29.7)	0.112	109 (26.7)	136 (40.7)	<0.001
Smoking (%)			0.893			
Current smoker	76 (13.9)	73 (14.3)		46 (11.3)	48 (14.4)	0.442
Former smoker	154 (28.3)	138 (27.0)		109 (26.7)	84 (25.1)	
Never smoker	315 (57.8)	301 (58.8)		253 (62.0)	202 (60.5)	
Intervention group (%)			0.224			0.059
Control	188 (34.5)	189 (36.9)		148 (36.3)	122 (36.5)	
MedDiet plus EVOO	201 (36.9)	163 (31.8)		156 (38.2)	104 (31.1)	
MedDiet plus nuts	156 (28.6)	160 (31.2)		104 (25.5)	108 (32.3)	

Data are shown as mean (SD) or N (%), as appropriate. Abbreviations: LTFA: leisure time physical activity; T2D: type 2 diabetes; CHD: coronary heart disease; MedDiet: Mediterranean diet; EVOO: extra-virgin olive oil; MET, metabolic equivalent of task. Duplicate controls, and those controls that were also cases during the follow-up, were removed.

**Table 2:** Association between baseline plasma glycolysis species or other related metabolites and incident atrial fibrillation.

Metabolite	Q1	Q2	Q3	Q4	P-Trend	Per 1-SD
Phosphoglycerate ^1^	122/188	141/182	132/179	117/185		512/734
Crude model	1 (Ref)	1.21 (0.86, 1.71)	1.06 (0.74, 1.52)	0.89 (0.62, 1.3)	0.4212	0.98 (0.86, 1.11)
Multivariate model	1 (Ref)	1.21 (0.85, 1.73)	1.05 (0.72, 1.52)	0.87 (0.59, 1.28)	0.3796	0.96 (0.84, 1.1)
Hexose monophosphate ^1^	135/177	120/177	122/191	131/182		508/727
Crude model	1 (Ref)	0.88 (0.62, 1.24)	0.8 (0.57, 1.14)	0.92 (0.65, 1.3)	0.6319	0.95 (0.84, 1.07)
Multivariate model	1 (Ref)	0.87 (0.61, 1.24)	0.79 (0.55, 1.15)	0.94 (0.65, 1.36)	0.7371	0.94 (0.82, 1.07)
Fructose/glucose/galactose ^1^	144/188	122/184	117/180	129/182		512/734
Crude model	1 (Ref)	0.84 (0.61, 1.17)	0.79 (0.57, 1.11)	0.9 (0.65, 1.25)	0.4974	1.00 (0.89, 1.13)
Model 2	1 (Ref)	0.83 (0.59, 1.18)	0.75 (0.51, 1.09)	0.94 (0.63, 1.42)	0.6463	1.03 (0.89, 1.19)
Lactate ^1^	113/184	135/181	120/188	144/181		512/734
Crude model	1 (Ref)	1.2 (0.85, 1.69)	1.13 (0.8, 1.60)	1.4 (0.98, 2.01)	0.0848	1.09 (0.97, 1.24)
Multivariate model	1 (Ref)	1.21 (0.85, 1.74)	1.1 (0.77, 1.59)	1.28 (0.88, 1.86)	0.2571	1.05 (0.92, 1.20)
Sucrose ^1^	125/185	118/182	132/190	137/177		512/734
Crude model	1 (Ref)	0.93 (0.67, 1.29)	1.04 (0.75, 1.46)	1.09 (0.78, 1.53)	0.5279	1.04 (0.92, 1.18)
Multivariate model	1 (Ref)	0.86 (0.61, 1.21)	1 (0.7, 1.42)	1.02 (0.71, 1.46)	0.7782	1.01 (0.89, 1.15)
α-glycerophosphate ^1^	125/179	114/184	146/186	127/184		512/733
Crude model	1 (Ref)	0.91 (0.64, 1.28)	1.25 (0.88, 1.77)	1.11 (0.77, 1.59)	0.3700	1.08 (0.95, 1.24)
Multivariate model	1 (Ref)	0.94 (0.66, 1.35)	1.27 (0.88, 1.83)	1.14 (0.78, 1.68)	0.3189	1.10 (0.96, 1.27)
PEP ^1^	99/162	111/160	98/155	125/154		433/631
Crude model	1 (Ref)	1.3 (0.88, 1.91)	1.13 (0.75, 1.68)	1.41 (0.94, 2.13)	0.1560	1.13 (0.98, 1.3)
Multivariate model	1 (Ref)	1.36 (0.89, 2.06)	1.15 (0.75, 1.75)	1.42 (0.91, 2.22)	0.1892	1.12 (0.96, 1.31)
Ratio PEP:lactate ^1^	98/158	94/157	122/162	119/154		433/631
Crude model	1 (Ref)	0.93 (0.63, 1.37)	1.25 (0.85, 1.83)	1.21 (0.81, 1.82)	0.2311	1.08 (0.94, 1.24)
Multivariate model	1 (Ref)	0.82 (0.54, 1.24)	1.27 (0.84, 1.92)	1.21 (0.78, 1.88)	0.2125	1.09 (0.94, 1.27)

Matched odds ratios are described (95% CI). Multivariate models: Multivariate models were adjusted for the intervention group (Mediterranean diet vs. control group), body mass index (kg/m^2^), smoking (current, former, or never), leisure time physical activity (metabolic equivalent tasks in min/d), prevalent chronic conditions at baseline (dyslipidemia, hypertension, and type 2 diabetes), family history of coronary heart disease, education level (primary or lower/secondary or higher), and medication for dyslipidemia, hypertension, and type 2 diabetes. ^1^, case/controls are described for each metabolite. Abbreviations: PEP: phosphoenolpyruvate; Ref: reference.

**Table 3:** Association between baseline plasma glycolysis species and other related metabolites and incident heart failure.

Metabolite	Q1	Q2	Q3	Q4	P-Trend	Per 1-SD
Phosphoglycerate ^1^	72/122	82/130	84/119	95/136		333/507
Crude model	1 (Ref)	1.08 (0.72, 1.63)	1.30 (0.83, 2.03)	1.25 (0.81, 1.93)	0.2808	**1.17 (1.00, 1.38)**
Multivariate model	1 (Ref)	1.30 (0.81, 2.1)	1.43 (0.86, 2.38)	1.54 (0.94, 2.54)	0.0975	**1.28 (1.06, 1.53)**
Hexose monophosphate ^1^	88/133	73/126	72/123	96/122		329/504
Crude model	1 (Ref)	0.87 (0.57, 1.35)	0.89 (0.58, 1.36)	1.24 (0.8, 1.9)	0.3519	1.08 (0.92, 1.27)
Multivariate model	1 (Ref)	0.93 (0.57, 1.53)	0.86 (0.54, 1.39)	1.16 (0.71, 1.88)	0.6258	1.05 (0.87, 1.25)
Fructose/glucose/galactose ^1^	73/133	77/119	90/134	94/122		334/508
Crude model	1 (Ref)	1.21 (0.8, 1.82)	1.26 (0.83, 1.9)	1.42 (0.96, 2.11)	0.0827	**1.17 (1.01, 1.35)**
Multivariate model	1 (Ref)	1.09 (0.68, 1.74)	0.88 (0.52, 1.49)	0.93 (0.53, 1.63)	0.7498	1.04 (0.85, 1.27)
Lactate ^1^	79/136	88/126	70/123	97/123		334/508
Crude model	1 (Ref)	1.12 (0.75, 1.67)	0.94 (0.59, 1.48)	1.39 (0.89, 2.18)	0.2012	1.08 (0.92, 1.27)
Multivariate model	1 (Ref)	1.00 (0.64, 1.56)	0.91 (0.55, 1.51)	1.13 (0.68, 1.86)	0.6855	1.00 (0.83, 1.20)
Sucrose ^1^	68/125	78/136	68/124	120/123		334/508
Crude model	1 (Ref)	1.09 (0.72, 1.66)	1.16 (0.73, 1.83)	**1.92 (1.26, 2.94)**	0.0014	**1.26 (1.08, 1.47)**
Multivariate model	1 (Ref)	1.01 (0.64, 1.61)	0.94 (0.56, 1.57)	1.57 (0.98, 2.52)	0.0461	1.18 (0.99, 1.40)
α-glycerophosphate ^1^	82/129	85/130	85/125	82/123		334/507
Crude model	1 (Ref)	1.15 (0.75, 1.77)	1.15 (0.74, 1.78)	1.19 (0.75, 1.88)	0.4946	1.02 (0.87, 1.2)
Multivariate model	1 (Ref)	0.98 (0.6, 1.62)	0.99 (0.6, 1.62)	1.23 (0.73, 2.08)	0.4054	1.04 (0.87, 1.25)
PEP ^1^	63/108	74/115	50/116	94/122		281/461
Crude model	1 (Ref)	0.93 (0.57, 1.51)	0.77 (0.47, 1.27)	1.12 (0.68, 1.82)	0.7114	1.07 (0.90, 1.28)
Multivariate model	1 (Ref)	0.62 (0.35, 1.10)	0.8 (0.46, 1.41)	0.89 (0.51, 1.56)	0.9817	1.03 (0.84, 1.25)
Ratio PEP:lactate ^1^	65/111	68/111	77/113	71/126		281/461
Crude model	1 (Ref)	1.05 (0.65, 1.68)	1.11 (0.7, 1.77)	0.91 (0.56, 1.5)	0.7976	1.02 (0.85, 1.21)
Multivariate model	1 (Ref)	0.91 (0.52, 1.61)	0.98 (0.58, 1.68)	0.89 (0.51, 1.57)	0.7650	1.03 (0.85, 1.26)

Matched odds ratios are described (95% CI). Multivariate models: Multivariate models were adjusted for the intervention group (Mediterranean diet vs. control group), body mass index (kg/m^2^), smoking (current, former, or never), leisure time physical activity (metabolic equivalent tasks in min/d), prevalent chronic conditions at baseline (dyslipidemia, hypertension, and type 2 diabetes), family history of coronary heart disease, education level (primary or lower/secondary or higher), and medication for dyslipidemia, hypertension, and type 2 diabetes. ^1^ case/controls are described for each metabolite. Abbreviations: PEP: Phosphoenolpyruvate; Ref: reference. Bold font indicates statistical significance.

**Table 4:** Association between baseline plasma glycolysis species (per 1-SD increase) and other related metabolites, and incident atrial fibrillation and heart failure, by diabetes status.

	Atrial Fibrillation		Heart failure	
Metabolite	Without Diabetes	With Diabetes	Without Diabetes	With Diabetes
Phosphoglycerate	0.89 (0.73, 1.07)	1.03 (0.87, 1.23)	0.95 (0.72, 1.25)	**1.57 (1.24, 1.98)**
Hexose monophosphate	0.90 (0.75, 1.09)	0.97 (0.81, 1.16)	0.83 (0.63, 1.1)	1.19 (0.95, 1.48)
Fructose/glucose/galactose	1.12 (0.88, 1.43)	0.99 (0.82, 1.19)	1.04 (0.73, 1.47)	1.01 (0.79, 1.28
Lactate	1.01 (0.85, 1.21)	1.10 (0.92, 1.31)	1.01 (0.77, 1.32)	1.01 (0.8, 1.27)
Sucrose	1.05 (0.88, 1.25)	0.97 (0.81, 1.17)	1.11 (0.87, 1.41)	1.18 (0.94, 1.48)
α-glycerophosphate	1.18 (0.97, 1.43)	1.04 (0.86, 1.26)	1.15 (0.87, 1.52)	0.95 (0.76, 1.18)
PEP	1.13 (0.93, 1.37)	1.11 (0.89, 1.38)	1.00 (0.76, 1.31)	1.10 (0.86, 1.4)
Ratio PEP:lactate	1.12 (0.92, 1.37)	1.05 (0.85, 1.29)	1.01 (0.76, 1.34)	1.09 (0.85, 1.39)

Matched odds ratios are described (95% CI). Multivariate models were adjusted for the intervention group (Mediterranean diet vs. control group), body mass index (kg/m^2^), smoking (current, former, or never), leisure time physical activity (metabolic equivalent tasks in min/d), prevalent chronic conditions at baseline (dyslipidemia, hypertension), family history of coronary heart disease, education level (primary or lower/secondary or higher), and medication for dyslipidemia, hypertension, and type 2 diabetes. Abbreviations: PEP: Phosphoenolpyruvate; Bold font indicates statistical significance.

## Data Availability

Data described in the manuscript, code book, and analytic code will not be made publicly available. PREDIMED data can be requested by signing a data sharing agreement as approved by the relevant research ethics committees and the steering committee of the PREDIMED trial (www.predimed.es (accessed on 1 April 2021)).
